# A case for absolute gene expression estimates in microbiome studies using metatranscriptomics

**DOI:** 10.1093/ismejo/wraf188

**Published:** 2025-08-22

**Authors:** Michiel Perneel, Harriet Alexander, Pascal I Hablützel, Steven Maere

**Affiliations:** Flanders Marine Institute (VLIZ), 8400 Oostende, Belgium; Department of Plant Biotechnology and Bioinformatics, Ghent University, 9052 Gent, Belgium; Center for Plant Systems Biology, VIB, 9052 Gent, Belgium; Biology Department, Woods Hole Oceanographic Institution, Woods Hole, MA, United States; Flanders Marine Institute (VLIZ), 8400 Oostende, Belgium; Biology Department, Vrije Universiteit Brussel, 1050 Brussel, Belgium; Department of Plant Biotechnology and Bioinformatics, Ghent University, 9052 Gent, Belgium; Center for Plant Systems Biology, VIB, 9052 Gent, Belgium

**Keywords:** metatranscriptomics, absolute gene expression, microbial community dynamics

Metatranscriptomics is widely used to study the functional dynamics of microbial communities in their natural environment [1–6]. Changes in gene expression in microbial communities can be subdivided in two main categories: *per capita* changes at the cellular or organismal scale in response to developmental cues and environmental signals, and changes mediated by population dynamics and taxonomic shifts. In this perspective, we argue that understanding the effects of population dynamics on gene expression patterns benefits from the estimation of absolute transcript abundances, augmenting the relative expression measures that are commonly used. We use a recent study on the seasonal dynamics of metabolic activity and species turnover of microeukaryotic surface plankton in the southern North Sea as an example [7].

## Use and drawbacks of relative expression measures in microbial metatranscriptomic studies

The number of RNA-Seq reads mapping to a given gene is not only determined by the gene’s expression in the sample analyzed, but also by other factors like sequencing depth (library size), transcript length (longer transcripts generate more reads [8]), and technical effects such as GC content bias [9]. Mapped read count data therefore need to be normalized to make gene expression values comparable within or across samples. Several methods have been proposed to normalize RNA-Seq read count data [10]. One of the most widely used methods currently is TPM (transcripts per million) normalization, where the mapped read counts are first normalized for transcript length and then scaled to sum to one million within each sample, accounting for sequencing depth. TPM normalization results in relative expression values (per million) that can be compared across genes within a sample and, under some conditions and assumptions (see below), across samples for the same gene. Due to its desirable comparability characteristics and its simplicity, TPM became the de facto standard normalization method for various kinds of downstream analyses on RNA-Seq data, including clustering, dimensionality reduction and data visualization.

Although TPM is a relative expression measure, it is frequently used as a proxy for absolute gene expression under the assumption that the total mRNA content (per unit volume) of the samples of interest is approximately the same. This assumption is often approximately valid when dealing with a set of samples of a specific cell type or tissue in a specific organism [11]. In other cases however, the relation of relative to absolute expression may be different for different samples [12]. A given tissue may highly express a particular set of genes as part of its developmental program, causing the fraction of other transcripts in the total mRNA pool to be lower. Consequently their relative expression is lower than in another tissue, even though these transcripts may be expressed at the same level in both tissues in absolute terms [13]. Alternatively, stress may induce global changes in absolute cellular mRNA levels. Heat shock treatment of primary human umbilical vein endothelial cells was found to result in an almost two-fold drop in absolute mRNA levels for the median gene after 12 h [14]. Large-enough differences in total mRNA content complicate the interpretation of relative gene expression differences across conditions or time points. It is well known for instance that such differences interfere with accurate detection of differentially expressed (DE) genes across conditions when using TPM or related relative expression measures based on within-sample normalization procedures [12]. In cases where the total mRNA content differences are caused by a limited set of genes that are very highly expressed in one condition versus another, the effects on relative expression and DE analysis can still be moderated by using between-sample normalization procedures such as those implemented in DESeq2 [15] or edgeR [16], which, in contrast to within-sample normalization procedures such as TPM, leverage the assumption that most genes are not DE across conditions [15, 16]. However, when the total mRNA content differences across conditions are more systematic, the compositional nature of RNA-Seq data entails that there are no algorithmic ways to infer an expression measure purely from the count data themselves that adequately reflects absolute expression differences across samples.

Differences in the total mRNA content of samples are ubiquitous in microbial metatranscriptomics. Samples from different ecosystems may contain vastly different amounts of mRNA depending on community composition, ecosystem history, and environmental conditions. But also within a single microbial ecosystem, the amount of mRNA per unit volume or weight (e.g. a liter of seawater or a gram of soil) frequently exhibits substantial temporal (e.g. seasonal, diel) and spatial variation [17]. One of the main causes of mRNA content differences between samples are differences in microbial density and biomass. The abundance of microbes in an ecosystem and their taxonomic composition change dynamically with resource availability and other ecological drivers. Moreover, even in samples with the same cell counts or biomass, metabolic and transcriptional activity and hence total mRNA content may differ depending on the environmental or physiological conditions organisms are experiencing. Relative expression measures such as TPM do not account for differences in microbial density and total mRNA content between samples, rendering these measures ill-suited to capture absolute changes in organismal or transcript abundances in dynamic microbial ecosystems. Nevertheless, TPM and other relative expression measures are routinely used in ecological studies [7, 18–23]. Relative expression measures are valuable tools to compare the relative abundance of a given transcript across samples, either relative to the total transcript pool or to the transcript pool of a particular microbial clade [21], or to compare the clade-specific relative abundance of transcripts between clades [18]. However, these measures should not inadvertently be misused as proxies for absolute transcript abundance in microbial ecosystem studies.

## Absolute expression measures for studying microbial population dynamics

To address the challenges posed by relative expression measures such as TPM for studying microbial ecosystem dynamics, absolute measures of gene expression such as TPL (transcripts per liter) have been proposed as an alternative [8, 24–26]. Absolute expression estimation relies on the addition of internal RNA standards to samples, which are either custom-built sets of synthetic RNA molecules of known concentration or commercially available RNA standards such External RNA Controls Consortium (ERCC) Spike-In mixes (Thermo Scientific), ArrayControl RNA Spikes (Invitrogen) or Sequins (Garvan Institute of Medical Research). These standards are added to samples either before RNA extraction or before library preparation. There should be enough internal standard added for effective quantification, but not too much so that it does not dominate the resulting reads [25]. As a rule of thumb, RNA standards should be added at a ratio of ⁓1% of the total extracted RNA [24]. If the RNA yield is approximately known, it is feasible and preferable to add standards before RNA extraction. However, when RNA yields vary unpredictably between samples, adding an adequate amount of RNA standard to each sample before extraction is challenging, and over- or underaddition of standard may jeopardize the sample. In such cases, either representative test samples can be sacrificed for estimating RNA yield in different groups of samples, or RNA standards can be added after RNA extraction and before library preparation instead [26]. In the latter case, RNA losses during extraction cannot be taken into account, rendering the resulting expression measures only approximately absolute. However, even when adding standards before RNA extraction, losses due to incomplete RNA extraction and differences in RNA extraction efficiency between different organisms in a sample (e.g. hard-shelled diatoms versus soft-bodied euglenids) cannot be corrected.

When spiking samples with internal RNA standards and documenting sample volumes throughout the RNA extraction, sample preparation, and sequencing steps, estimating the absolute number of transcripts in the original sample volume becomes possible. Calculation of absolute expression levels is generally a two-step process, where first TPM values are calculated for both the internal standards and the transcripts of interest, and then TPM values are converted to TPL (or similar measures such as transcripts per cell or per gram of soil) using a conversion factor per sample estimated from the TPM values of the internal standards and the known quantities in which they were added to the sample. Estimation of this conversion factor is preferably done using a generalized linear model that simultaneously models the different standards added to the sample in different quantities [27–29], but sometimes an average of the conversion factors calculated for each internal standard separately is used instead [7]. The resulting TPL estimates facilitate the comparison of absolute rather than relative expression values across samples.

Just like TPM estimates, TPL estimates are not immune to biases. Research on the efficiency of internal RNA standards used for absolute expression normalization indicates that the standards can be subject to library preparation effects and that the proportion of reads they produce in a sequencing run may deviate in a sample-specific manner from the nominal proportion at which they were spiked into the RNA sample [30]. Next to the aforementioned compositional bias caused by variable RNA extraction efficiencies across organisms, TPL and TPM values can also be biased due to e.g. differences in microbe filtering or isolation efficiency during sampling or differences in RNA degradation within and across samples during storage. These biases warrant due caution in the interpretation the resulting expression profiles. Achieving reliable expression quantification, whether relative or absolute, requires minimal technical variation in sampling and sample processing. We therefore encourage researchers to safeguard the quality and reproducibility of their sampling and RNA processing workflows. In the early days of microbial metatranscriptomics, when technical variability in sample processing was all but unavoidable, focusing on relative expression measures such as TPM may have been the safest option because relative measures based on within-sample normalization are less sensitive to across-sample technical variability in e.g. global RNA extraction efficiency than absolute measures. However, as the reproducibility of workflows for RNA extraction and processing has improved substantially in recent years, it has become feasible to reliably measure absolute expression profiles across large sets of microbial ecosystem samples.

## Case study: The use of relative versus absolute expression measures to study population dynamics in the southern North Sea

Recently, we performed a year-long metatranscriptomics-based survey of micro-eukaryotic plankton dynamics in the southern Bight of the North Sea [7]. Here, we use some of the data from this study to highlight the limitations of relative expression measures such as TPM when studying the functional dynamics of microbial communities over time. Our metatranscriptomics approach involved filtering the 50–250 μm organismal size fraction from 50 L of seawater sampled monthly at different stations, extracting all RNA from these samples, adding ERCC Spike-In mix to each sample after extraction, and sequencing the mRNA fraction [7]. After mapping the reads to a reference metatranscriptome that was assembled de novo from the sequencing data at all stations throughout the year, TPM values for each transcript in each sample were estimated using Kallisto [31]. In parallel, we used the ERCC Spike-In mix to estimate absolute TPL expression levels, taking into account the filtered volume of seawater, sample processing volumes, and the extracted and processed amounts of RNA [7] (as we only added ERCCs after RNA extraction, any differences in RNA extraction efficiency between samples remain unaccounted for in the resulting TPL estimates).

**Figure 1:**
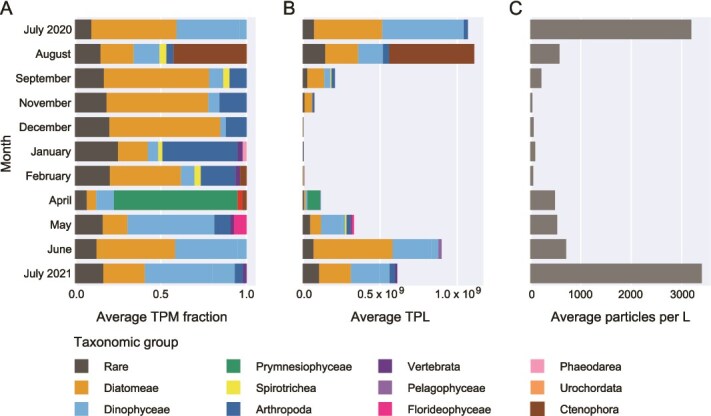
Monthly biomass fluctuations and turnover in taxonomic composition. (A) Monthly relative transcript abundance (TPM fraction) of high-level taxonomic groups annotated using EukProt (>60% sequence identity with reference), averaged across sampling stations. The TPM fraction of a group in a given month was calculated as the sum of TPMs for transcripts annotated to that group divided by the TPM sum over all groups for that month (excluding unannotated transcripts). When the TPM fraction of a group was <2%, it was labeled as “rare”. (B) Monthly estimates for the number of transcripts per liter of sea surface water (TPL), averaged across sampling stations. Transcripts were annotated using EukProt (>60% sequence identity with reference) and averaged across stations. (C) Monthly number of particles per liter of seawater, averaged across stations, estimated using FlowCam automated image analysis (excluding unannotated particles).

**Figure 2:**
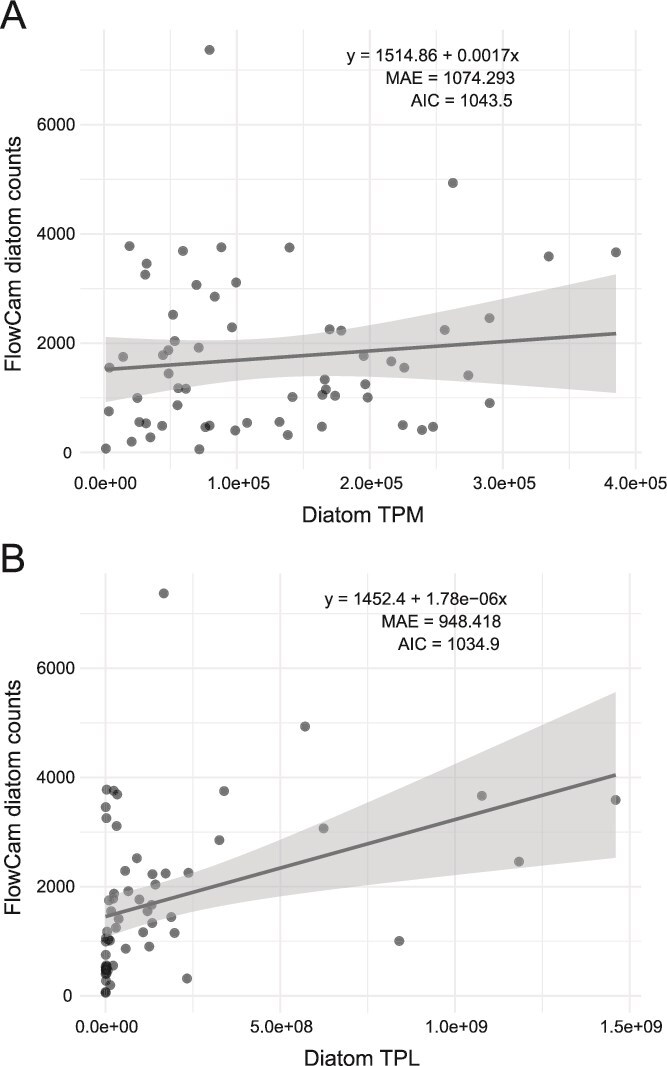
Comparison of diatom TPM and TPL with FlowCam-derived diatom counts. (A) Linear regression of FlowCam diatom counts on diatom TPM (*R*^2^ = 0.013, AIC = 1043.5). (B) Linear regression using TPL (*R*^2^ = 0.146, AIC = 1034.9). Shaded ribbons in panels (A) and (B) represent 95% confidence intervals.

TPM and TPL values paint complementary but qualitatively different pictures of the seasonal microbial dynamics in the southern North Sea (Fig. 1). Interpretation of seasonal dynamics based on TPM profiles show a substantially higher relative expression of diatom transcripts in September–December relative to August, suggesting a rise in their ecological relevance and perhaps abundance in autumn (Fig. 1). However, the TPL and FlowCam profiles reveal that the increase in diatom relative expression is accompanied by a significant decrease in the absolute abundance of diatoms and other microeukaryotes, which leads to very different conclusions regarding the state of the ecosystem. Similarly, diatoms may appear less abundant or transcriptionally active in April than in February or May based on the TPM expression profiles, but the TPL profiles reveal that the reduced diatom TPM levels in April are rather due to a bloom of a haptophyte species (*Phaeocystis globosa*) that temporarily dominates the RNA pool (Fig. 1). These examples highlight one of the most important drawbacks of TPM: TPM values do not adequately reflect changes in community composition and overall microbial abundance over time, rendering their interpretation from a population dynamics perspective ambiguous and misleading. TPM normalizes transcript counts within a sample, meaning that when one taxon becomes more abundant or transcriptionally active, the TPM values of all other taxa are reduced, even if their absolute abundance and activity remain constant. In contrast, TPL more accurately reflects ecological changes. In contrast to diatom TPM values, diatom TPL values show a significant linear association with FlowCam-derived diatom counts (Fig. 2, TPM regression slope *P* = 0.3824, TPL regression slope *P* = 0.0026). This shows that absolute expression levels are more closely related to absolute organism abundances than relative expression levels are, because the latter are skewed by the presence of other organisms in the ecosystem. Whereas the difference between TPM and TPL spatial or temporal profiles is expected to be less pronounced in studies targeting ecosystems with less variability in biomass, total RNA content, and taxonomic composition than the seasonal study of a temperate coastal sea presented here, the limitations of TPM as a proxy for absolute expression are likely relevant to at least some extent for most microbial ecosystems. However, also the relationship between TPL values and FlowCam counts is far from perfect, in part because non-metabolically active diatom silica shells are also recognized as diatoms by the automated imaging classifier. In this respect, TPL values may sometimes give a more accurate picture of absolute organism abundances than FlowCam counts.

**Figure 3:**
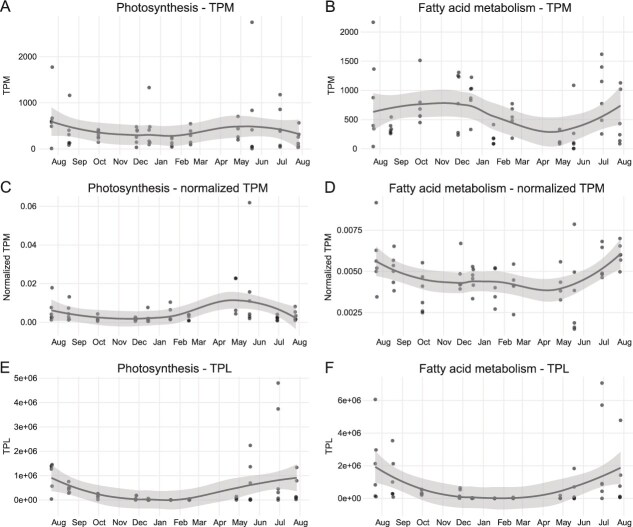
Temporal dynamics of diatom metabolism across expression metrics. LOESS-smoothed expression trends are shown for two key metabolic functions in diatoms: photosynthesis and fatty acid metabolism. Rows represent (A–B) TPM profiles, (C–D) TPM profiles normalized to the total diatom TPM per sample (normalized TPM), and (E–F) TPL profiles for photosynthesis (left panels) and fatty acid metabolism (right panels). TPM and TPL values are summed per sample for diatom-assigned transcripts (percent sequence identity >60%) annotated to the metabolic function concerned. Normalized TPM values represent the contribution of each metabolic function to the total diatom transcript pool in each sample. Smoothed lines represent LOESS fits with 95% confidence intervals indicated as shaded ribbons.

TPL reflects seasonal trends better than TPM not only for microbiome composition, but also for molecular process activity (Fig. 3). The TPM profile for diatom photosynthesis for instance suggests that photosynthetic activity in diatoms is fairly constant throughout the year. In contrast, the TPL profile shows virtually no photosynthetic activity in winter and more pronounced peak activity in summer, as expected. The discrepancy between the TPM and TPL profiles arises because the TPM values in autumn and winter are inflated by the overall higher relative abundance of diatoms in these seasons (Fig. 1). In many process-specific diatom TPM profiles, e.g. for fatty acid biosynthesis (Fig. 3B), this effect causes a pronounced increase in autumn and winter that disappears in the corresponding TPL profiles (Fig. 3F). When normalizing the process-specific TPM profiles to the total diatom TPM levels in each month, this effect largely disappears, but also the interpretation changes. Whereas the photosynthesis TPM profile for example shows the diatom photosynthetic transcriptional activity relative to the total transcriptional activity in the sample across time, and the TPL profile shows the absolute photosynthetic transcriptional activity, the normalized TPM profile shows the photosynthetic transcriptional activity relative to the total diatom transcriptional activity, i.e. the relative transcriptional investment of diatoms in photosynthesis. TPL and normalized TPM values hence convey different information. In summary, TPL estimates facilitate the comparison of gene expression values in microbial communities across spatiotemporal scales and more accurately reflect absolute organism abundances.

## Conclusion and recommendations

Although TPM is currently the *de facto* standard for quantifying gene expression in metatranscriptomics studies, it is not well suited for spatiotemporal monitoring of dynamic microbial ecosystems. As a relative expression measure based on within-sample normalization, TPM does not adequately capture absolute changes in organismal or transcript abundances across samples, making it ill-suited to account for natural fluctuations in population size and metabolic activity – and this limitation persists when comparing specific genes, metabolic features, or the taxonomic composition of microbial communities across space and time. Although taxon-specific normalization of TPM values can address relative expression biases caused by the variable presence or activity of other taxa, and thereby provides a more accurate view on the relative transcriptional investments of a taxon across samples, taxon-normalized TPM values are still relative expression measures that do not account for changes in the absolute abundance or transcriptional activity of the taxon of interest. Absolute expression measures such as TPL, despite having limitations of their own, are better suited for between-sample comparisons and can provide ecological insights complementary to the knowledge gained from relative expression analysis [32]. We therefore advocate for the use of internal RNA standards when profiling metatranscriptomes of complex microbial ecosystems. Not only do these internal standards allow for comparison of absolute expression levels across samples in a spatiotemporal dataset, but they may also facilitate comparisons across datasets and improve our ability to couple gene expression profiles to biogeochemical measurements, which is crucial to obtain an integrated understanding of the dynamics and functioning of microbial communities in natural environments.

## Data Availability

The raw metatranscriptomics data reanalyzed in this perspective are available on SRA as BioProject PRJNA1021244 (https://www.ncbi.nlm.nih.gov/bioproject/PRJNA1021244). The resulting preprocessed metatranscriptomics data and the FlowCam count data are available on Zenodo (https://doi.org/10.5281/zenodo.15837363). The analysis scripts are available on GitHub (https://github.com/MichielPerneel/absolute-metatranscriptomics).
